# Continuous blood flow visualization with laser speckle contrast imaging during neurovascular surgery

**DOI:** 10.1117/1.NPh.9.2.021908

**Published:** 2022-03-07

**Authors:** David R. Miller, Ramsey Ashour, Colin T. Sullender, Andrew K. Dunn

**Affiliations:** a The University of Texas at Austin, Department of Biomedical Engineering, Austin, Texas, United States; b The University of Texas at Austin, Dell Medical School, Department of Neurosurgery, Austin, Texas, United States

**Keywords:** laser speckle contrast imaging, neurovascular surgery, blood flow imaging, cerebral blood flow, indocyanine green angiography

## Abstract

**Significance:**

Laser speckle contrast imaging (LSCI) has emerged as a promising tool for intraoperative cerebral blood flow (CBF) monitoring because it produces real-time full-field blood flow maps noninvasively and label free.

**Aim:**

We aim to demonstrate the ability of LSCI to continuously visualize blood flow during neurovascular procedures.

**Approach:**

LSCI hardware was attached to the surgical microscope and did not interfere with the normal operation of the microscope. To more easily visualize CBF in real time, LSCI images were registered with the built-in microscope white light camera such that LSCI images were overlaid on the white light images and displayed to the neurosurgeon continuously in real time.

**Results:**

LSCI was performed throughout each surgery when the microscope was positioned over the patient, providing the surgeon with real-time visualization of blood flow changes before, during, and after aneurysm clipping or arteriovenous malformation (AVM) resection in humans. LSCI was also compared with indocyanine green angiography (ICGA) to assess CBF during aneurysm clipping and AVM surgery; integration of the LSCI hardware with the microscope enabled simultaneous acquisition of LSCI and ICGA.

**Conclusions:**

The results suggest that LSCI can provide continuous and real-time CBF visualization without affecting the surgeon workflow or requiring a contrast agent. The results also demonstrate that LSCI and ICGA provide different, yet complementary information about vessel perfusion.

## 1 Introduction

Cerebral blood flow (CBF) monitoring is routine during cerebrovascular surgery to inform decision-making.^1^ In cerebral aneurysm clipping cases, various technologies are routinely used to confirm patency in vessels and determine successful aneurysmal obliteration. Current intraoperative tools for CBF monitoring and visualization include indocyanine green angiography (ICGA),^2^^–^^7^ Doppler^8^^–^^10^ and transit-time^11^^,^^12^ ultrasound, and percutaneous transfemoral digital subtraction angiography (DSA).^13^^–^^17^ ICGA records the fluorescence wash in of a bolus of indocyanine green (ICG) after intravenous injection. DSA images are acquired by obtaining multiple time-controlled x-rays as contrast medium is injected intra-arterially.

During cerebrovascular procedures, it would be beneficial to assess CBF continuously, instead of a limited number of times.^18^ ICGA is an effective decision-making aid; however, it cannot provide continuous imaging as it requires an injected contrast agent. Doppler ultrasound provides absolute flow velocities, but is limited to measurement at single locations, and requires contact with the vessel of interest. DSA is the gold standard for confirming aneurysmal occlusion and patency of the underlying parent vasculature; however, it is invasive and time-consuming relative to ICGA or Doppler ultrasound, usually requiring removal of the surgical microscope, fluoroscopy, and transfemoral selective arterial catheterization.

Laser speckle contrast imaging (LSCI) has emerged as a promising tool to noninvasively monitor CBF because it produces real-time, full-field blood flow maps without any contrast agents, providing a potential continuous CBF monitoring solution. Both LSCI and ICGA images are restricted to the tissue surface, but their relatively simple instrumentation allows them to be incorporated into the surgical microscope. Several studies have demonstrated LSCI during neurosurgical procedures in humans and shown its promise as a CBF monitoring tool, including surgical revascularization,^19^^–^^21^ awake functional mapping,^22^ brain tumor resection,^23^^–^^25^ cortical spreading depression,^26^ and infarction during ischemic stroke.^27^ However, many previous clinical implementations required an external device to be introduced into the surgical field^19^^,^^20^^,^^27^ leading to disruptions of the surgical procedures. Other implementations incorporated the instrumentation into the neurosurgical operating microscope, eliminating the need for an external device and surgical disruption.^23^^–^^25^ However, LSCI imaging still required the surgical procedure to be paused while images were acquired, and it was not possible to record CBF images for long durations, or simultaneously with ICGA.

Here we demonstrate an LSCI system that is integrated into the neurosurgical microscope allows real-time, continuous visualization of CBF overlayed onto the surgical field during neurosurgical procedures including simultaneous ICGA and LSCI imaging. We evaluate these capabilities during cerebral aneurysm clipping and arteriovenous malformation (AVM) resection surgeries. Although simultaneous imaging of CBF with LSCI and ICGA has been performed in animal models,^28^^,^^29^ this comparison has not been performed in humans. This paper demonstrates the potential of LSCI for human CBF monitoring in two ways: LSCI was performed continuously during cerebral aneurysm clipping and AVM resection surgeries without affecting the surgical workflow, including real-time visualization of CBF during aneurysm clip placement, and LSCI and ICGA were performed simultaneously to visualize CBF for n=5 neurovascular cases. Taken together, these results demonstrate that LSCI can monitor CBF continuously during neurovascular procedures when the LSCI device is integrated into the surgical microscope and that LSCI and ICGA provide different yet complementary information about vessel perfusion.

## 2 Materials and Methods

### 2.1 Instrumentation

**Fig. 1:**
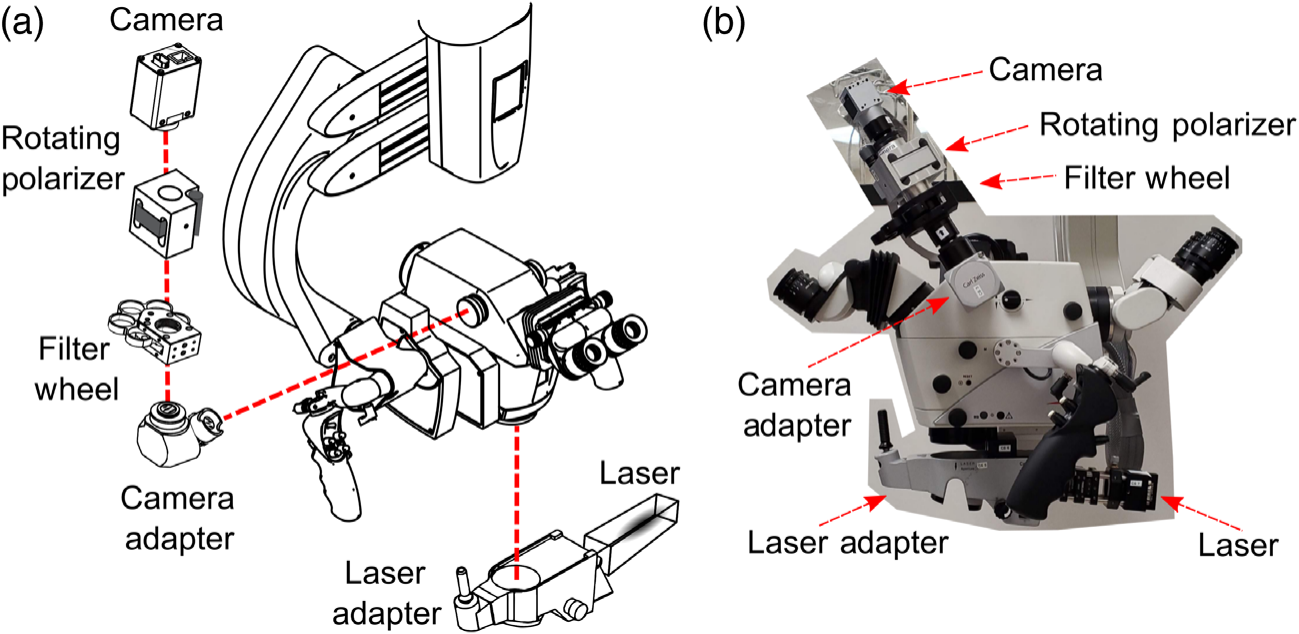
(a) Schematic of the Leica OH6 neurosurgical microscope outfitted with LSCI instrumentation to intraoperatively measure cerebral blood flow. Drawings adapted from Leica M530 OH6 Brochure and Thorlabs Inc. filter wheel diagram. (b) Photograph of the LSCI hardware attached to the Leica OH6 microscope in the operating room.

The LSCI hardware^24^ was attached to the microscope prior to the start of the surgery and did not interfere with the sterile draping or normal operation of the microscope. A schematic of the LSCI setup adapted to a surgical microscope (Leica M530 OH6, Leica Microsystems GmbH, Wetzlar, Germany) is shown in Fig. 1. A λ=785  nm laser diode with a maximum output power of 300 mW was attached to an add-on laser adapter (MM6 Micromanipulator, Carl Zeiss Meditec Inc., Oberkochen, Germany). The laser adapter was mounted to the bottom of the microscope such that a steering mirror directed the light downward toward the surgeon’s field of view. The beam size was ∼2  cm at a working distance of 35 cm. The maximum irradiance was $0.10\;W/{\mathrm{cm}}^{2}$, well below the American National Standards Institute limit of $0.3\;W/{\mathrm{cm}}^{2}$ for skin at 785 nm.^30^

Back-scattered laser light was directed to an NIR-enhanced CMOS camera (Basler AG, Ahrensburg, Germany) mounted on the side observer port on the same side as the craniotomy. This enabled an observer to participate during the study, which occurred for most of the surgery for patients 2 and 3 and intermittingly for patients 1, 4, and 5. The pixel area was slightly cropped during acquisition to capture only pixels over brain tissue. A filter wheel (CFW6, Thorlabs Inc.) and polarizer (LPNIR100, Thorlabs Inc.) were positioned between the camera adapter and camera. The filter wheel held various neutral density filters for controlling the laser power. The polarizer was integrated into a motorized rotation mount (RSC-100, Pacific Laser Equipment Inc., Santa Ana, California, USA) to reduce specular reflections. A band-pass filter (FF01-788/3-25, Semrock Inc., Rochester, New York, USA) was added in front of the camera to enable simultaneous LSCI acquisition during illumination of ICG, to block nonlaser light, and to avoid interference of normal white light illumination throughout each procedure.

### 2.2 Image Analysis

ICGA images are represented as raw fluorescence intensity images that were collected by the built-in Leica OH6 fluorescence camera. Raw LSCI images collected by the speckle camera were processed by calculating the spatial speckle contrast value (K) within a 7×7  pixel moving window according to the equation $K=\frac{\sigma_{s}}{I}$, where $\sigma_{s}$ is the spatial standard deviation and I  is the average intensity within the region. For relative blood flow measurements, speckle contrast was converted into correlation time ($\tau_{c}$) by evaluating the average decay time of the speckle electric field autocorrelation function^31^ for which we assumed unity for the instrumentation factor. The speckle correlation time $\tau_{c}$ is a more quantitative measure of blood flow; the inverse correlation time ($\mathrm{ICT}=1/\tau_{c}$) is commonly used as a metric for blood flow in vessels or perfusion in parenchyma.^32^^–^^35^ For displaying ICT time course data, the first 5 s of data were used as a normalization factor to more easily visualize the change in the flow relative to the baseline value. To overlay LSCI with images from the built-in microscope white light camera, the video output of the surgical microscope system was recorded continuously. LSCI images, white light reflectance images, and ICGA images were spatially co-registered by applying an affine transformation to all corresponding images.

**Fig. 2:**
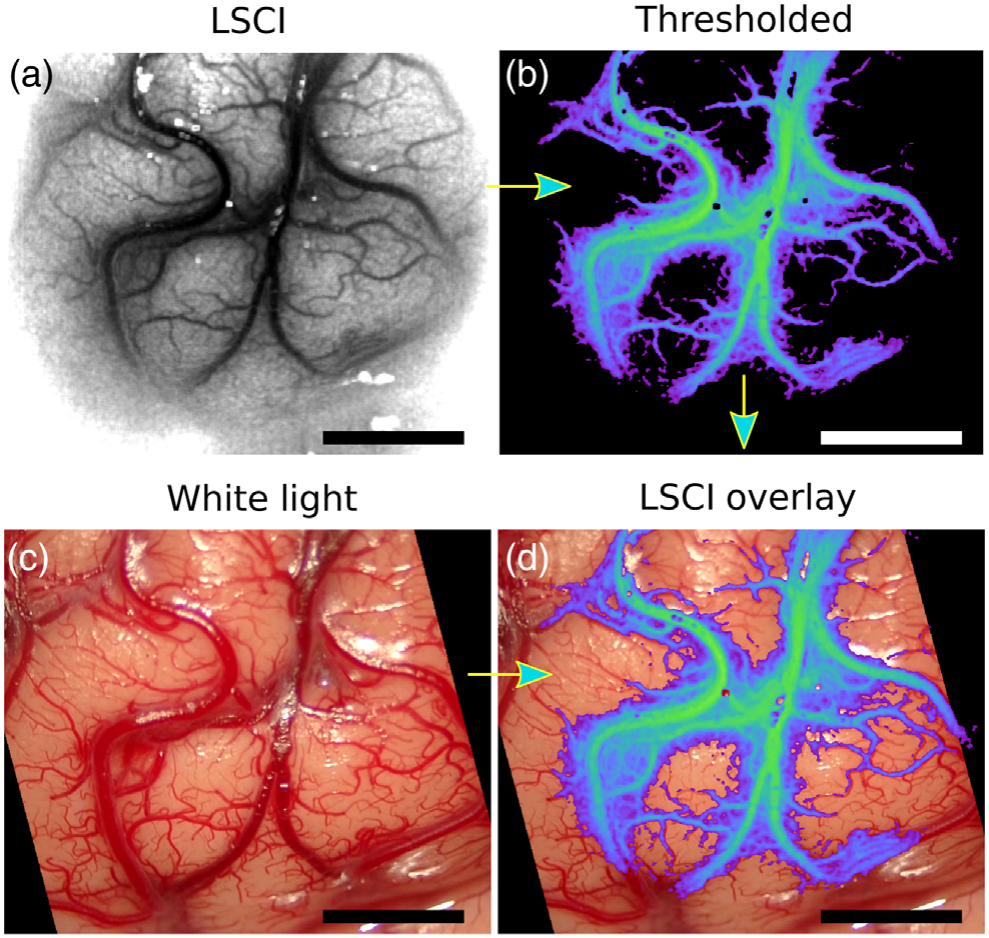
Image processing steps to create overlay of LSCI on the built-in microscope white light camera termed LSCI overlay. Data from patient 1 were used to create images. (a) LSCI image with grayscale color map. (b) LSCI image with a threshold applied to display high-flow blood vessels with median filter and pseudocolor applied. (c) White light image captured at the same time as LSCI image. (d) Thresholded pseudocolor LSCI image merged with the white light image to create an LSCI overlay image. Scale bars are 0.5 cm.

To better visualize CBF in real time, LSCI images were thresholded and overlaid on the visible white light reflectance images, as illustrated in Fig. 2. First, the LSCI image was acquired, shown in Fig. 2(a) with a grayscale color map. Next, a threshold was applied to the LSCI image such that only speckle contrast values corresponding to flow values within a certain range remain (i.e., high flow in vessels); additionally, a median filter and pseudocolor were applied for easier visualization, as shown in Fig. 2(b). Following the threshold and pseudocolor, the LSCI image was merged with the white light image [Fig. 2(c)] along with desired transparency [Fig. 2(d)]. Processing the LSCI frames, thresholding the high-flow values, registering the thresholded LSCI image with the white light image, and overlaying the thresholded LSCI image onto the white light image to create the overlay image were performed with custom software on a desktop PC at video rate and displayed continuously to the neurosurgeon on a monitor in real time.

### 2.3 Intraoperative Procedure

All n=5 surgeries were performed at Dell Seton Medical Center at the University of Texas at Austin by the neurosurgical co-investigator on this study (R. A.). The clinical study was approved by the Institutional Review Board of the University of Texas at Austin and performed in accordance with relevant guidelines and regulations. Written and informed consent was obtained from all patients prior to surgery. A summary of patient details is shown in Table S1 in the Supplementary Material.

Prior to surgery, the field of view of the camera used for LSCI was co-aligned and centered with the built-in microscope camera. Additionally, the laser beam was centered with the built-in microscope camera field of view. After the craniotomy was performed, the microscope was positioned over the patient at the discretion of the neurosurgeon (R. A.). LSCI could be performed at any time when the microscope was positioned over the patient by turning on the laser illumination. LSCI did not disturb the workflow of the neurosurgeon and was performed at numerous critical times throughout the surgery. The neurosurgical co-investigator (R. A.) performed a majority of the surgeries using the surgical microscope oculars and could observe the LSCI data in real time on a monitor mounted next to the surgical microscope that displayed the overlaid blood flow images.

## 3 Results

**Fig. 3:**
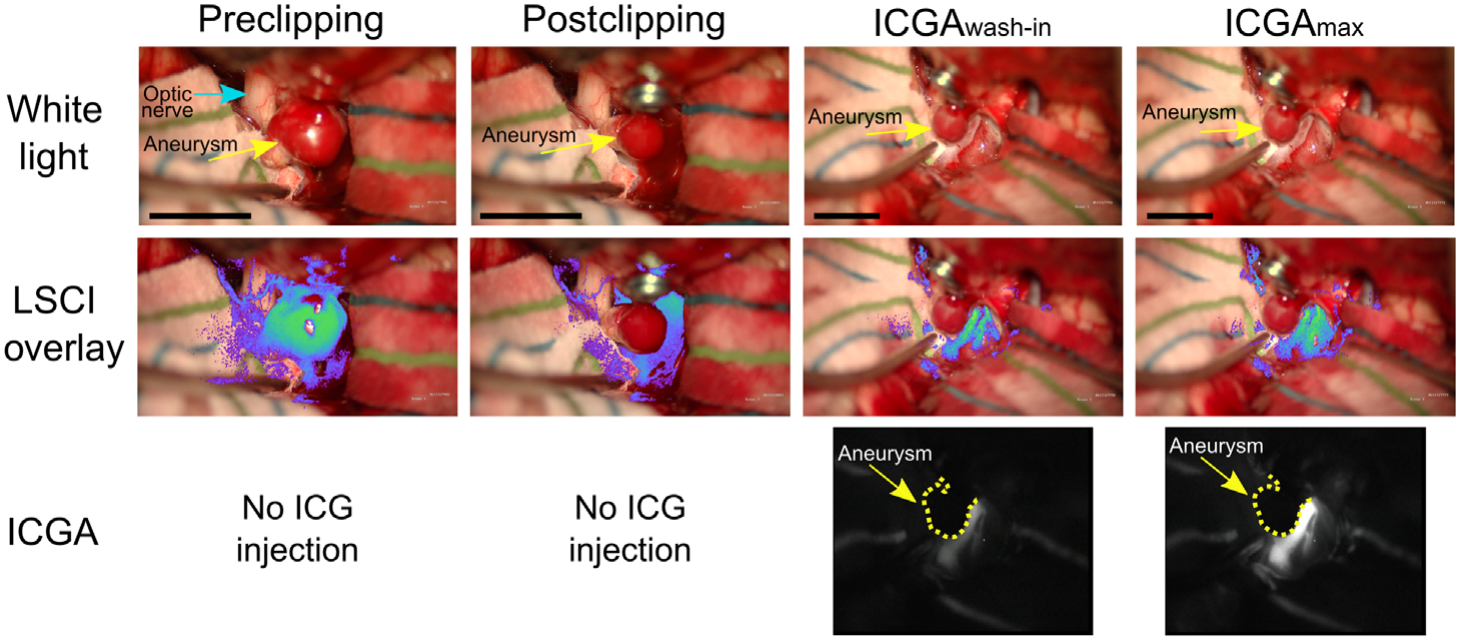
Images acquired from patient 2 before aneurysm clipping (preclipping), immediately after aneurysm clipping (postclipping), and during ICGA at the wash-in of the dye (${\mathrm{ICGA}}_{\mathrm{wash}\text{-}\text{in}}$) and at maximum fluorescence signal (${\mathrm{ICGA}}_{\text{max}}$). Visible light images were acquired from the built-in microscope white light camera (white light); LSCI images were acquired by an NIR-enhanced CMOS camera adapted to the microscope; and ICGA images were acquired by the built-in microscope NIR camera. LSCI overlay images were created by thresholding LSCI images and overlaying them onto the white light image with pseudo-color. Scale bars are 1 cm. Video 1 shows a montage of the white light and LSCI overlay images before, during, and after the clipping procedure (Video 1, MPEG, 19.8 MB [URL: https://doi.org/10.1117/1.NPh.9.2.021908.1]). Video 2 shows a montage of the white light, LSCI overlay, and ICGA images during the injection of the ICG dye (Video 2, MPEG, 18.2 MB [URL: https://doi.org/10.1117/1.NPh.9.2.021908.2]).

Figure 3 shows images from single time points preclipping and postclipping of the aneurysm and during ICGA for patient 2. White light and LSCI images were acquired throughout the duration of the aneurysm clipping, and ICGA images are only available during the injection of the ICG dye after the aneurysm clip was placed. In this surgery, a temporary clip was placed on the patient’s carotid artery in the neck after the preclipping images but before the aneurysm clipping.

The preclipping LSCI images in Fig. 3 show that there is high flow within the aneurysm prior to clipping and that LSCI has the spatial resolution to visualize the flow on the small vessels on the optic nerve (identified by the blue arrow on the preclipping white light image in Fig. 3). Video 1 further illustrates that LSCI can visualize the filling of the aneurysm during the cardiac cycle and the pulsatile motion of the flow within the aneurysm. This is particularly evident at the timestamp of 11 s in Video 1 when the temporary clip is placed on the patient’s carotid artery in the neck, causing a reduction of the flow in the aneurysm. An advantage of having the LSCI instrumentation integrated into the microscope, as opposed to as a stand-alone device, is that images can be acquired during operation of the microscope without interrupting the surgical workflow. This advantage is illustrated in Video 1 at a timestamp of 59 s, at which point the clip is placed on the aneurysm and the LSCI blood flow map immediately shows that there is a cessation of the flow in the aneurysm. This is similarly illustrated in the postclipping LSCI image in Fig. 3. Approximately 5 min after the clipping, ICGA is used to confirm successful aneurysmal obliteration and patency in surrounding vessels. In Fig. 3, the ICGA image during maximum fluorescent signal (${\mathrm{ICGA}}_{\text{max}}$) reveals cessation of the flow in the aneurysm.

**Fig. 4:**
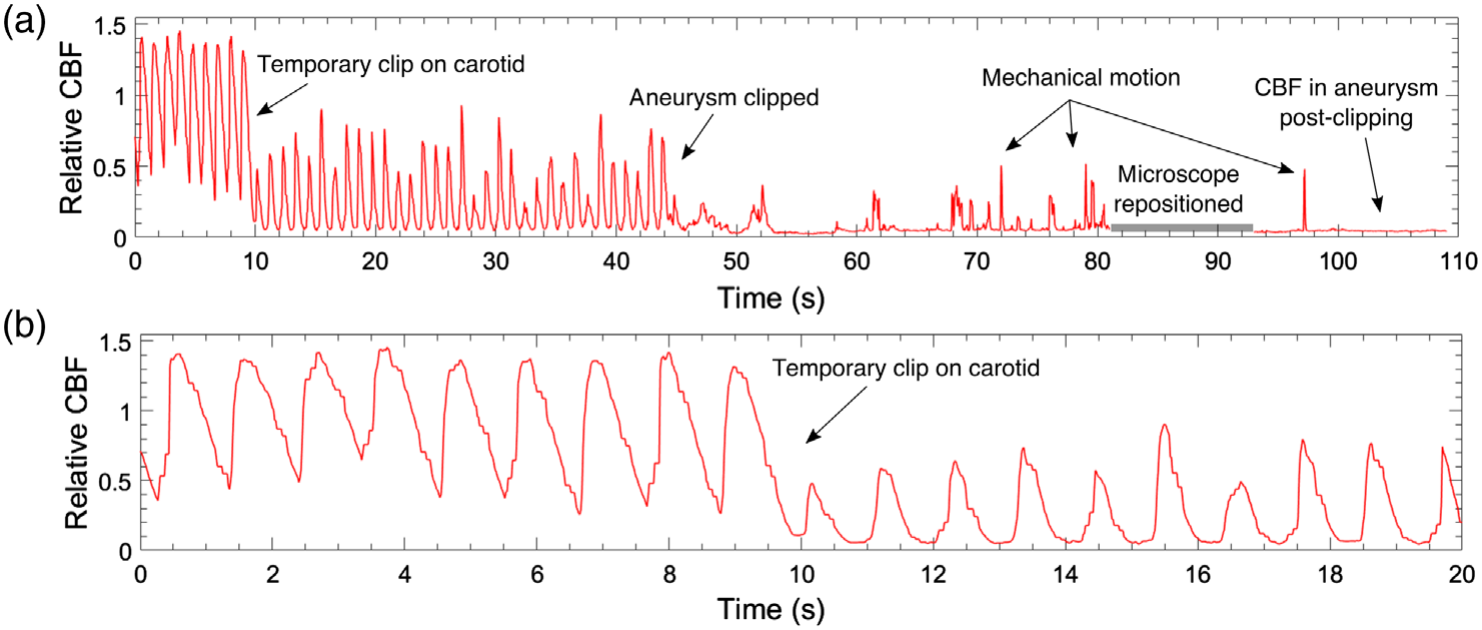
Time courses of the relative CBF within the aneurysm from patient 2 during the clipping procedure (time matches are shown in Video 1) for (a) 110 s and (b) zoomed in to the first 20 s. The relative CBF is normalized to the first five seconds of the data. The pulsatile nature of the flow in the aneurysm is clearly visible before and after the temporary clip is placed on the carotid artery. The reduction of CBF after the carotid temporary clip is immediately evident. Postclipping cessation of the flow is also clear. All of the transients after aneurysm clipping (t=45  s) are motion artifacts.

The flow dynamics within the aneurysm shown in Video 1 are quantified in Fig. 4. The pulsatile flow in the aneurysm is visible before and after the temporary clip is placed on the carotid artery in the neck. There is about a 70% reduction in average CBF after the temporary clip is placed on the carotid, and the pulsatile flow within the aneurysm is clearly visible with nulls of <5% and variable peaks of 50% to 80% of baseline. After the clip is placed on the aneurysm, there is a significant reduction in the flow and complete disappearance of pulsatile flow. The transient spikes after the clip placement are due to the surgeon mechanically pushing on the aneurysm; LSCI is sensitive to motion from both blood flow and mechanical force. After the microscope is repositioned, it is obvious that the CBF within the aneurysm is absent and is within the lower limit of single exposure LSCI measurements^36^ (∼4% of the initial CBF).

**Fig. 5:**
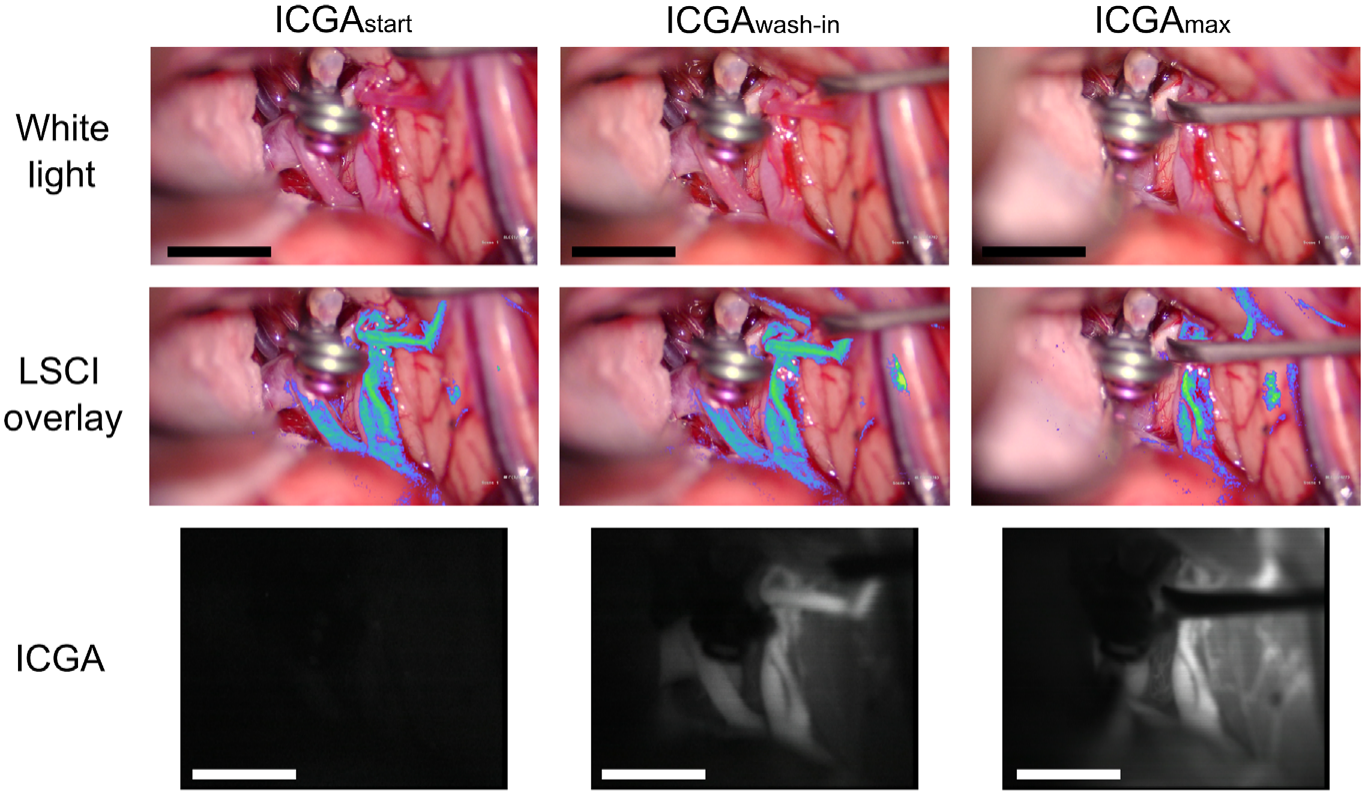
Images acquired from patient 4 during ICGA at the start of the injection (${\mathrm{ICGA}}_{\text{start}}$), at wash-in of the dye (${\mathrm{ICG}}_{\mathrm{wash}\text{-}\text{in}}$), and at maximum fluorescence signal (${\mathrm{ICGA}}_{\text{max}}$). Video 3 shows a montage of the white light, ICGA, and LSCI blood flow images during the entire ICGA procedure for which green pseudocolor is used for the LSCI overlay (Video 3, MPEG, 6.1 MB [URL: https://doi.org/10.1117/1.NPh.9.2.021908.3]). Visible light images were acquired from the built-in microscope white light camera (white light); LSCI images were acquired by an NIR-enhanced CMOS camera adapted to the microscope; and ICGA images were acquired by the built-in microscope NIR camera. LSCI overlay images were created by thresholding LSCI images and overlaying them onto the white light image. Scale bars are 1 cm.

Figure 5 shows images from single time points during ICGA following the first aneurysm clipping procedure for patient 4, which required two clips.

**Fig. 6:**
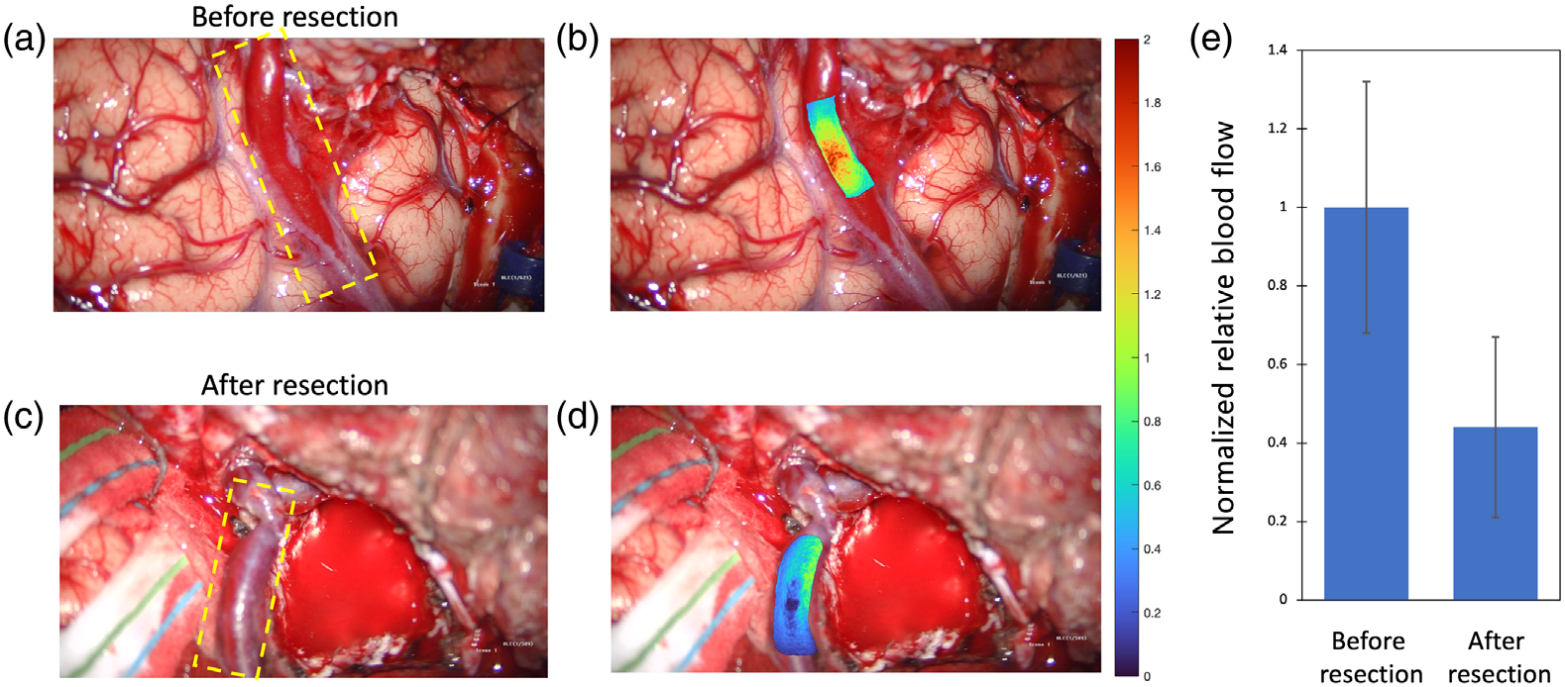
Images during the AVM resection from patient 5. (a) White light image showing the draining vein (outlined in yellow) of the AVM before the AVM is resected. (b) LSCI overlay depicting the relative blood flow in the draining vein before the AVM resection. The relative blood flow is normalized by the average blood flow in the highlighted region of interest. (c) White light image showing the draining vein (outlined in yellow) after the AVM resection. (d) LSCI overlay depicting the relative blood flow in the draining vein after the AVM resection. The relative blood flow is normalized by the average blood flow in the highlighted region of interest in (b) to compare the relative change in blood flow between (b) and (d). The color bar and color scale apply to both (b) and (d). (e) Plot of the normalized relative blood flow values in the draining vein before the AVM resection (b) and after the AVM resection (d).

Figure 6 demonstrates the ability of LSCI for long-term monitoring of the flow in the draining vein of the AVM [outlined in yellow in Figs. 6(a) and 6(c)] for patient 5. The white light image in Fig. 6(a) shows the draining vein of the AVM before the AVM is resected. Figure 6(b) shows the relative blood flow overlaid on the white light image for the same time point as Fig. 6(a); the blood flow values have been converted to the inverse correlation time and then normalized by the mean value within the highlighted region of interest in the draining vein. Figure 6(c) shows the white light image after the AVM resection taken 2 h and 40 min following the time point of Figs. 6(a) and 6(b). Figure 6(d) shows the relative blood flow overlaid on the white light image for the same time point as Fig. 6(c); the blood flow values have been converted to inverse correlation time and then normalized by the mean value within the highlighted region of interest in the draining vein in Fig. 6(b) to compare the relative blood flow changes before and after the AVM resection. Thus the color bar and color scale to the right of Figs. 6(b) and 6(d) apply to both Figs. 6(b) and 6(d). Figure 6(e) plots the normalized relative blood flow values in the draining vein from before the AVM resection shown in Fig. 6(b) and after the AVM resection shown in Fig. 6(d). The mean normalized relative blood flow in the draining vein is 1.0 for Fig. 6(b) and 0.4 for Fig. 6(d) with a standard deviation of 0.3 and 0.2, respectively. The 60% reduction in blood flow in the draining vein after the AVM is resected suggests that that blood flow is no longer bypassing capillary networks through the AVM.

**Fig. 7:**
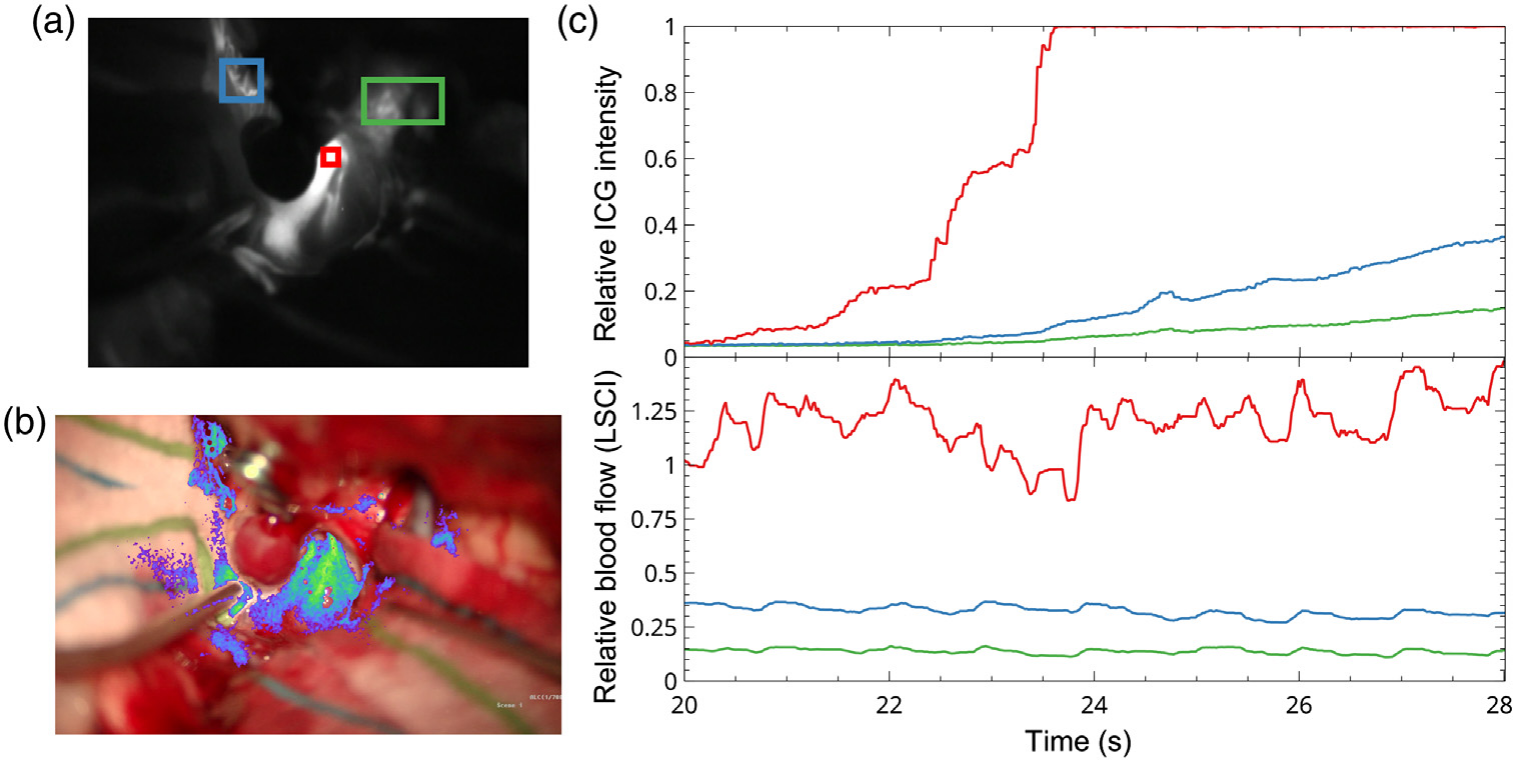
Time course of the fluorescence intensity change during bolus administration of ICG and the LSCI measures of relative blood flow for the same regions of interest. (a) ICG angiography image during wash-in of the ICG dye. Three regions of interest, for which the time course of the fluorescence intensity is shown in (c), are highlighted in red, green, and blue. (b) LSCI overlay image at the same time as the ICGA image in (a). The three regions of interest selected from the LSCI overlay displayed in (c) are the same regions as highlighted in (a). (c) Time course of the changes in the ICG fluorescence intensity (top) and relative blood flow measured by LSCI (bottom) during bolus administration of ICG. The three regions of interest are color coded to correspond with the respective images in (a) and (b).

Figure 7 compares the information obtained with LSCI and ICGA. ICGA intensity is a more direct measure of cerebral blood volume (CBV), whereas LSCI is directly sensitive to motion and therefore is a more direct measure of CBF. ICGA wash-in can also be used to identify feeding versus draining vessels in some surgical procedures, and the temporal dynamics of the fluorescence intensity can be used to estimate CBF.^37^^,^^38^ Figure 7 shows the time course of the fluorescence intensity change during bolus administration of ICG along with the LSCI measures of relative blood flow for the same regions of interest. In this example, the ICG fluorescence signal saturates in the largest vessel, but it is still clear that the rise time of the ICG signal is more rapid in this vessel than in the two smaller vessels, indicating a higher flow. The LSCI signals from the same regions reveal similar information but in a different manner. The LSCI signals are steady state because they are direct measures of the flow, which is relatively constant over the measurement period. However, the relative flow across each region of interest is evident from the steady-state values of the speckle decorrelation times. Therefore, although LSCI is currently unable to quantify absolute CBF, it can be used to estimate relative CBF over time or across spatial regions.

**Fig. 8:**
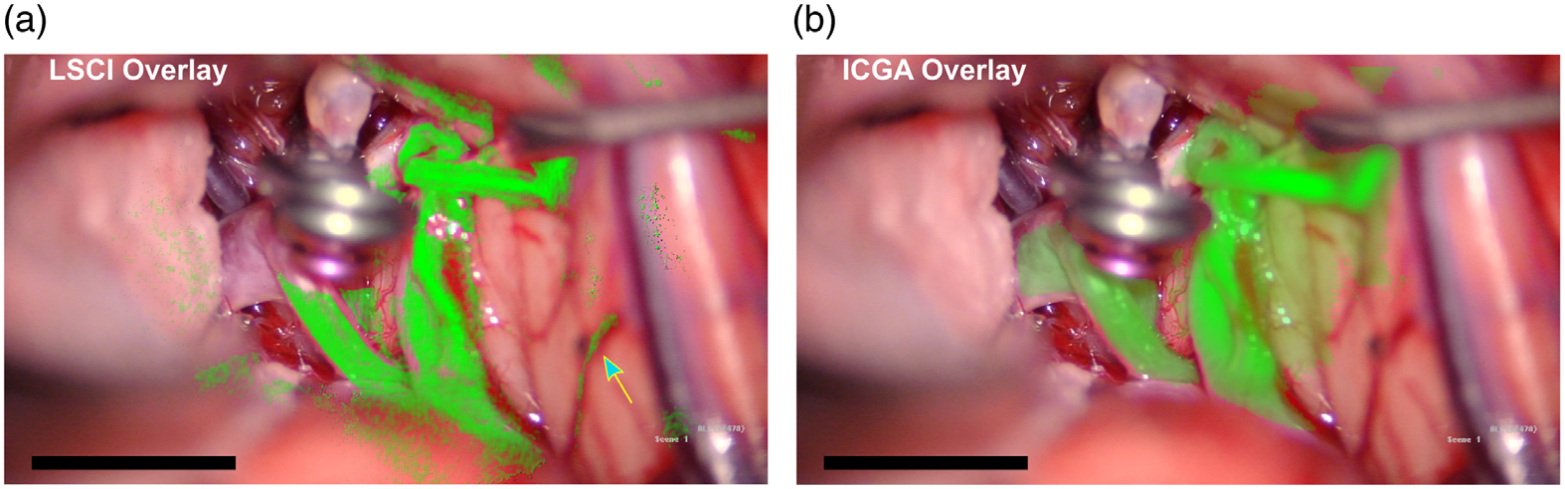
A comparison of (a) LSCI overlay with (b) ICGA overlay from patient 4. The images were created by overlaying the LSCI data and ICGA data, respectively, onto the built-in microscope white light camera and applying green pseudocolor. The arrow in (a) highlights LSCI’s ability to detect blood flow in side wall vessels. Scale bars are 1 cm.

Despite the differences between the information obtained with LSCI and ICGA, LSCI can also be used to create images that look very similar to ICGA images. Results comparing simultaneous LSCI and ICGA images shown in Fig. 8 and Video 3 demonstrate the complementary nature of information provided by LSCI during ICGA. The LSCI and ICGA images show similar spatial information when rendered with similar green color maps and overlaid onto the surgeon’s view.

## 4 Discussion

Results of the current study show that LSCI can be used to continuously monitor CBF in real time. Figure 3 and Video 1 demonstrate LSCI’s ability to continuously monitor CBF during critical parts of neurosurgical procedures when integrated into the surgical microscope and overlaid onto the surgeon’s view of the surgical field. LSCI reveals high flow in the aneurysm before the aneurysm clipping, and immediately after clipping, the aneurysm flow ceases while the surrounding vessels maintain perfusion. ICGA is then used to confirm patency in the surrounding vessels, but it requires administration of a dye. This demonstrates that LSCI is complementary to ICGA; both can be used to determine perfusion in vessels within the surgical field of view.

Results shown in Fig. 4 demonstrate that LSCI enables quantification of the flow in the aneurysm relative to a baseline value. LSCI can detect the pulsatile flow profile within the aneurysm before the aneurysm is clipped. After clipping, LSCI shows that there is a >96% reduction of the flow in the aneurysm relative to the initial flow and no pulsatile flow. We estimate the uncertainty in the relative flow for LSCI measurements to be ∼5% since LSCI is sensitive to any form of motion within the tissue;^36^ thus the aneurysm may be fully occluded, yet the LSCI signal will not reduce by 100% from baseline. It is expected that the LSCI signal would still be sensitive to Brownian motion of scattering particles within the aneurysm; however, this signal will be significantly smaller than flow from red blood cells into the aneurysm. Although further work is needed to determine the percentage reduction of the flow for a surgeon to be confident that the aneurysm is fully occluded, Fig. 4 offers preliminary evidence that a 96% reduction in the flow in an aneurysm as measured by LSCI indicates successful aneurysmal obliteration.

The waveforms in Fig. 4 depicting the CBF within the aneurysm match those measured with Doppler ultrasonography^39^ and a Doppler velocity wire.^40^ Figure 4 highlights that LSCI allows for continuous CBF measurements within an aneurysm throughout neurosurgery, and thus LSCI may be useful for improving our understanding of the hemodynamics in aneurysms and validating computational fluid dynamic models of the growth and rupture of aneurysms.^41^^,^^42^

Figure 6 demonstrates the ability of LSCI for long-term monitoring of the flow in a surface vessel during AVM resection. Figures 6(a), 6(c), and 6(e) show that the surface vessel next to the AVM is initially arterialized and then the flow is reduced after the AVM resection. This demonstration shows the LSCI has the potential to quantify relative changes in the blood flow within feeding and draining vessels in real time over the course of an AVM resection, which can take several hours, thus providing vital and actionable information to the surgeon on the success of the surgery. Future work will aim at establishing the repeatability of such flow measurements when the surgical environment changes.

Figure 7 depicts how the ICGA intensity signal is a measure of CBV whereas LSCI is a measure of CBF. However, LSCI images can be processed to appear similar to ICGA images, as shown in Fig. 8. ICGA is better able to visualize flow in larger vessels due to ICGA using fluorescent dye as a contrast, whereas LSCI uses the inherent properties of the blood flow to scatter laser light. Conversely, LSCI has an advantage in visualizing the flow in small vessels, as shown in Fig. 8 on the blood vessels in the side walls marked by the blue arrow and in Fig. 3 for which LSCI shows CBF in small vessels supplying the optic nerve. ICGA also has the advantage of providing the directionality of the flow during the wash-in of the dye.

One limitation of LSCI that is noticeable in Fig. 4, Videos 1-2 (linked in Fig. 3), and Video 3 (linked in Fig. 5) is that LSCI is sensitive to motion from both the blood flow and the surgeon’s mechanical force on the brain tissue. Thus the LSCI overlay videos cannot differentiate between the two sources of motion. However, the motion induced by the surgeon can be avoided by the surgeon pausing for a moment and viewing the LSCI overlay while not pushing on the tissue. We note that the integration of the hardware for this study is not optimal, and image quality could be improved with better hardware integration such as integrating both the laser and camera internally to the microscope. This would allow for optimization of light collection by reducing the number of optics that the collected light travels through before reaching the camera.

The aim of this study was to demonstrate the potential of LSCI to monitor CBF continuously during neurovascular surgery. The integration of the LSCI device into the surgical microscope enabled continuous CBF visualization and allowed for simultaneous acquisition of LSCI and ICGA. The time course of the relative blood flow shown in Figs. 4 and 7 and the LSCI overlay view shown in Videos 1-2 (linked in Fig. 3) and Video 3 (linked in Fig. 5) demonstrate the ability of LSCI to monitor CBF continuously and in real time. The sequence of simultaneous LSCI and ICGA images shown in Figs. 3, 5, 7, and 8 suggests that LSCI and ICGA provide complementary information about CBF as both can be used to determine perfusion in a vessel.

## 5 Conclusion

Our results suggest that LSCI can provide continuous and real-time CBF visualization without affecting the surgeon workflow or requiring a contrast agent and thus is a promising tool for continuous CBF monitoring during surgery. By integrating the LSCI device into the surgical microscope, we performed LSCI at critical parts of neurovascular surgery and provided the surgeon with immediate actionable information on the success of the procedure. We also performed simultaneous acquisition of LSCI and ICGA, demonstrating that LSCI and ICGA are complementary tools for visualizing CBF to aid surgical decision making.
