# Supplementary material for: Continuous blood flow visualization with laser speckle contrast imaging during neurovascular surgery
Source: Neurophotonics. 2022 Mar 7;9(2):021908. doi: 10.1117/1.NPh.9.2.021908 (PMC8900813; doi:10.1117/1.NPh.9.2.021908)
Supplement: Supplementary file 1 [file NPh_009_021908_SD001.docx]

**Continuous blood flow visualization with laser speckle contrast imaging during neurovascular surgery**

**David R. Miller, PhD,^1^ Ramsey Ashour, MD,^2^ Colin T. Sullender, PhD,^1^ and Andrew K. Dunn, PhD^1,*^**

^1^Department of Biomedical Engineering, The University of Texas at Austin, Austin, TX, USA

^2^Department of Neurosurgery, Dell Medical School, The University of Texas at Austin, Austin, Texas, USA

**1 Supplementary Materials**

**1.1** *Patient Information*

Patient 1: A woman between the ages of 35 and 45 with a family history of fatal cerebral aneurysm rupture (aunt) presented with headaches and was found to have a 5 mm wide-necked lobulated right superior cerebellar artery aneurysm on computed tomography angiography (CTA). Cerebral angiography was performed, and the aneurysm was deemed unfavorable for endovascular repair. While observation with serial imaging was discussed based on her unruptured status and the deep location of the aneurysm, surgery was also offered in light of her youth and family history. She was taken to the operating room (OR) for clipping of the aneurysm, with complete aneurysmal occlusion and patency of the underlying vasculature confirmed by intraoperative cerebral angiography. She had an expected postoperative ipsilateral oculomotor palsy due to dissection and mobilization of the nerve during the surgery, which resolved at 3 months follow-up.

Patient 2: A woman between the ages of 60 and 70 with an incidental right ophthalmic segment cerebral aneurysm diagnosed years prior on brain imaging obtained after a fall presented to establish care. Follow-up CTA and subsequent cerebral angiography were performed, demonstrating a lobulated right paraclinoid aneurysm with an 11 mm superiorly projecting dome. Given the size, morphology, and demonstrated growth of the aneurysm, treatment was recommended. An initial attempt at Pipeline embolization was aborted due to significant pre-procedural bleeding from her endotracheal tube and arterial line site. In spite of reducing her clopidogrel dose to ½ tablet every other day, follow-up platelet aggregation testing revealed a persistent supratherapeutic response to clopidogrel, and she was thus referred for open surgical clipping. She was taken to the OR for clipping of the aneurysm. Clinoidectomy was deferred due to extensive pneumatization of the clinoid. Occlusion of the aneurysm’s intracranial component and patency of the underlying vasculature was confirmed by intraoperative cerebral angiography. She made an uneventful recovery without complication at 6 months follow-up.

Patient 3: A woman between the ages of 75 and 85 presenting with headaches was found to have a wide-necked lobulated 11 mm left middle cerebral artery aneurysm, deemed unfavorable for endovascular repair. She was taken to the operating room for uneventful surgical clipping, with complete aneurysmal occlusion confirmed on intraoperative cerebral angiography. She made an uneventful recovery without complication.

Patient 4: A woman between the ages of 50 and 60 with hypertension was admitted for stroke-like symptoms and found on magnetic resonance angiography (MRA) to have 4 cereb­ral aneurysms: a 7 mm right middle cerebral artery aneurysm, a 6 mm anterior communicating artery aneurysm, a 5 mm left pericallosal artery aneurysm, and a 5 mm left middle cerebral artery aneurysm. She was brought to the operating for right craniotomy and clipping of the right middle cerebral and anterior communicating artery aneurysms in the same setting. Intraoperative cerebral angiogram after clipping showed successful occlusion of the clipped aneurysms with patency of the underlying vasculature. She had transient postop confusion and had to be readmitted for evacuation of a subgaleal pseudomeningocele and epidural fluid collection. She ultimately did not require a shunt and recovered back to her baseline by 2 months postop.

Patient 5: A woman between the ages of 35 and 45 with temporomandibular joint pain underwent imaging of her jaw and was incidentally found to have a right temporal arteriovenous malformation (AVM). This was further evaluated with brain MRA and cerebral angiography, demonstrating a high-flow right temporal AVM with a compact 2 cm nidus supplied by middle cerebral artery branches with early venous drainage into a markedly enlarged right middle cerebral vein with more minor venous outflow via subtemporal veins. She underwent craniotomy for AVM resection which was uneventful. Intraoperative angiography confirmed complete angiographic obliteration of the AVM. Follow-up brain MRA 3 months postoperatively demonstrated no residual or recurrent AVM. She remains neurologically intact.

A summary of patient details is shown in Supplemental Table 1.

**Supplemental Table 1. Patient details for each aneurysm case.**

| Patient | Gender | Age | Procedure Location/Size | Preop Imaging | # times ICGA | Intraop and Postop Imaging | Clinical Outcome |
| --- | --- | --- | --- | --- | --- | --- | --- |
| 1 | F | 35-45 | Right SCA 5mm aneurysm | CTA  DSA | 1 | Intraop DSA  Postop CTA | Transient CN III palsy resolved |
| 2 | F | 60-70 | Right ICA 11mm aneurysm | CTA  DSA | 2 | Intraop DSA | No complication |
| 3 | F | 75-85 | Left MCA 11mm aneurysm | MRI  DSA | 1 | Intraop DSA | No complication |
| 4 | F | 50-60 | Right MCA and ACOM aneurysm | MRA  DSA | 2 | Intraop DSA | Transient cognitive dysfunction resolved |
| 5 | F | 35-45 | Right temporal lobe AVM | MRA  DSA | 1 | Intraop DSA  Postop MRA | No complication |

ACOM = anterior communicating artery; AVM = arteriovenous malformation; CN = cranial nerve; CTA = computed tomography angiography; DSA = digital subtraction angiography; ICA = internal carotid artery; ICGA = indocyanine green angiography; MCA = middle cerebral artery; MRA = magnetic resonance angiography; SCA = superior cerebellar artery.

Example angiographic images of patient’s cerebral aneurysm(s) are shown in Supplemental Fig. 1.


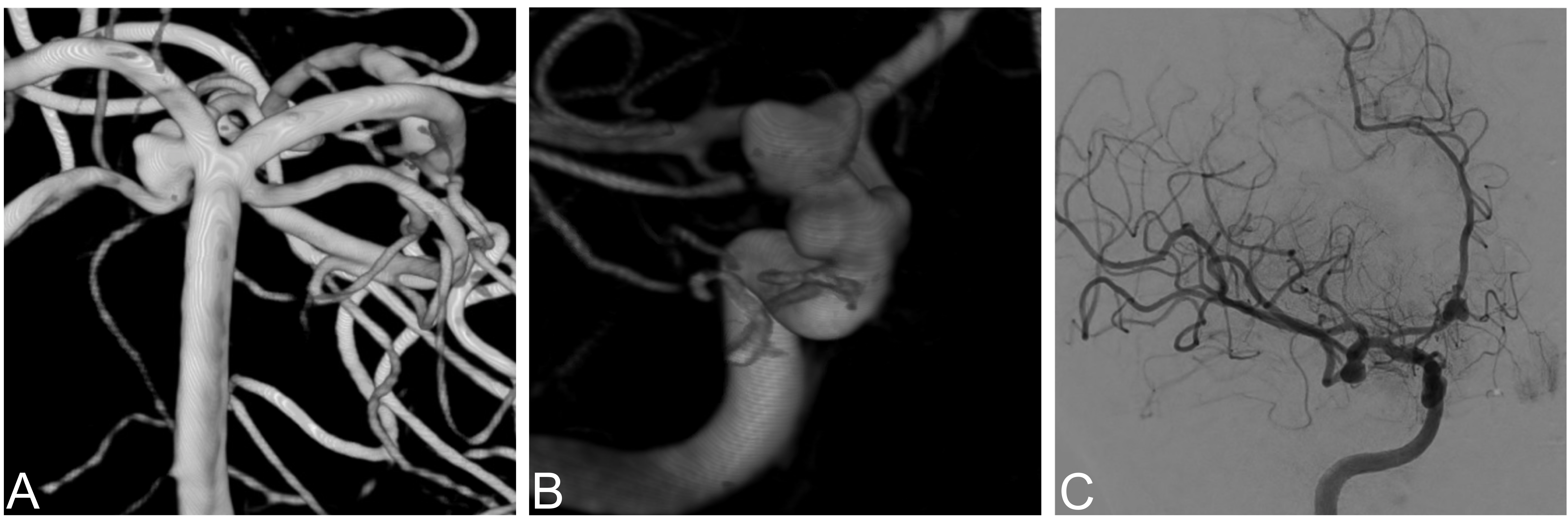


**Supplemental Fig. 1** Angiographic images of cerebral aneurysm clipping cases. **A**: Image from Patient 1 with a 5 mm wide-necked lobulated right superior cerebellar artery aneurysm. **B**: Image from Patient 2 showing a lobulated right paraclinoid aneurysm with an 11 mm superiorly projecting dome. **C**: Image from Patient 4 with 4 cerebral aneurysms: a 7mm right middle cerebral artery aneurysm, a 6 mm anterior communicating artery aneurysm, a 5 mm left pericallosal artery aneurysm, and a 5 mm left middle cerebral artery aneurysm.
